# Identification of Metabolomic Signatures for Ischemic Hypoxic Encephalopathy Using a Neonatal Rat Model

**DOI:** 10.3390/children10101693

**Published:** 2023-10-16

**Authors:** Yulia Shevtsova, Chupalav Eldarov, Natalia Starodubtseva, Kirill Goryunov, Vitaliy Chagovets, Oleg Ionov, Egor Plotnikov, Denis Silachev

**Affiliations:** 1 V.I. Kulakov National Medical Research Center for Obstetrics Gynecology and Perinatology, Ministry of Healthcare of Russian Federation, 117997 Moscow, Russia; yu_shevtsova@oparina4.ru (Y.S.); ch_eldarov@oparina4.ru (C.E.); n_starodubtseva@oparina4.ru (N.S.); k_gorunov@oparina4.ru (K.G.); v_chagovets@oparina4.ru (V.C.); o_ionov@oparina4.ru (O.I.); plotnikov@belozersky.msu.ru (E.P.); 2 A.N. Belozersky Institute of Physico-Chemical Biology, Lomonosov Moscow State University, 119992 Moscow, Russia

**Keywords:** metabolomics, lipidomics, diagnostics, neonatal asphyxia, liquid chromatography–mass spectrometry

## Abstract

A study was performed to determine early metabolomic markers of ischemic hypoxic encephalopathy (HIE) using a Rice–Vannucci model for newborn rats. Dried blood spots from 7-day-old male and female rat pups, including 10 HIE-affected animals and 16 control animals, were analyzed by liquid chromatography coupled with mass spectrometry (HPLC-MS) in positive and negative ion recording modes. Multivariate statistical analysis revealed two distinct clusters of metabolites in both HPLC-MS modes. Subsequent univariate statistical analysis identified 120 positive and 54 negative molecular ions that exhibited statistically significant change in concentration, with more than a 1.5-fold difference after HIE. In the HIE group, the concentrations of steroid hormones, saturated mono- and triglycerides, and phosphatidylcholines (PCs) were significantly decreased in positive mode. On the contrary, the concentration of unsaturated PCs was increased in the HIE group. Among negatively charged molecular ions, the greatest variations were found in the categories of phosphatidylcholines, phosphatidylinositols, and triglycerides. The major metabolic pathways associated with changed metabolites were analyzed for both modes. Metabolic pathways such as steroid biosynthesis and metabolism fatty acids were most affected. These results underscored the central role of glycerophospholipid metabolism in triggering systemic responses in HIE. Therefore, lipid biomarkers’ evaluation by targeted HPLC-MS research could be a promising approach for the early diagnosis of HIE.

## 1. Introduction

Hypoxic-ischemic encephalopathy (HIE) is a condition that occurs when the oxygen supply and blood flow to the brain of a newborn are decreased [1]. This reduction in oxygenation and blood flow can be caused by a variety of factors, including complications during labor and delivery, problems with the umbilical cord, abnormalities of the placenta, or maternal health conditions. HIE is a common cause of neonatal mortality and disability in newborns. The incidence of HIE is estimated to be 2–6 per 1000 live births [2]. HIE is responsible for 6–9% of all neonatal deaths and 21–23% of deaths in full-term infants, while 25% develop severe neurological impairments such as cerebral palsy, seizures, intellectual disability, cognitive impairment, and epilepsy [3,4,5]. These conditions, which are often irreversible or difficult to treat, present significant challenges for those affected and their families. The lifelong impact of these disabilities underscores the importance of early diagnosis, rapid intervention, and comprehensive rehabilitative care for children with HIE.

The incidence of HIE has not decreased despite advances in obstetric care such as fetal monitoring to prevent hypoxia-ischemia. Nevertheless, most of the current neonatal studies of HIE aim to minimize the extent of subsequent brain injury. In the past, treatment options were limited to drug therapy to maintain cardiopulmonary function and relieve convulsive activity. However, in recent years, therapeutic hypothermia (TH) has become an established and effective treatment approach for HIE [6]. It has been shown to improve survival and long-term outcomes in affected children [7,8]. There are several mechanisms that make TH effective in the treatment of HIE. First, it helps to reduce the levels of free radicals and glutamate, which can cause further damage to the brain. In addition, hypothermia lowers oxygen demand, helping to minimize the effects of the initial hypoxic-ischemic insult. Finally, it attenuates cellular apoptosis, thus reducing brain damage [9]. For optimal outcomes, TH should ideally be initiated within the first 6 h of life. Early intervention is critical for maximizing the potential benefits of hypothermia treatment [10]. Unfortunately, despite the proven benefits of therapeutic hypothermia, it is discouraging to note that approximately 20% of neonates who eventually develop moderate-to-severe HIE because a brain injury was not diagnosed in a timely manner do not receive the necessary clinical attention and miss the opportunity to undergo TH [11].

The method of TH also has a number of limitations, and its use can be dangerous if the diagnosis is incorrect [12,13,14]. Therefore, correct diagnosis of HIE in neonates is a key factor in initiating treatment with TH. Therefore, there is an urgent need to identify reliable markers for early diagnosis of neonates with perinatal asphyxia who later develop encephalopathy [15]. Clinical studies in the neonatal period are very limited. Noninvasive samples (urine, stool) and minimally invasive samples (blood, dried blood spots) are advantageous in studying the dynamics of the molecular composition of biological fluids. After routine clinical examinations, a certain number of such samples remain that can be used for further investigations without the need to collect additional biological material. However, the scientific task of searching for metabolic markers for the development of HIE is complicated by the small number of patients with this nosology, the impact of asphyxia on organs and systems other than the brain, and the presence of combined pathologies (e.g., sepsis), which is reflected in the alteration of the integral metabolic profile of blood plasma [16]. Thus, in most cases, when analyzing the blood plasma of patients, it is possible to obtain a set of metabolites that reflect various pathological processes in the neonate’s body. To search for metabolomic markers associated only with brain damage, studies are performed on model systems. The data obtained in this way can be taken into account in the analysis and interpretation of the clinical results of the study to search for new molecular markers of ischemic-hypoxic brain damage. Pattaraporn Chun and colleagues performed intrauterine umbilical cord occlusion in *Macaca nemestrina* primates to induce HIE and obtained cord blood samples for two-dimensional gas chromatography with mass spectrometry (GC-MS). The analysis revealed a number of metabolites that could be potential biomarkers, including arachidonic acid, butanoic acid, citric acid, fumaric acid, lactate, malate, propanoic acid, and succinic acid [17]. In turn, Julia Kuligowski and a group of colleagues performed metabolomic studies of blood plasma in a model of hypoxia-ischemia in newborn piglets. Hypoxia-ischemia was achieved by ventilating the lungs with a gas mixture containing 8% O_2_. Blood samples were collected before the onset of hypoxia, at the end of hypoxia, and 120 min after the end of hypoxia for subsequent analysis by liquid chromatography–mass spectrometry. The results showed a maximum correlation of the levels of choline, 6,8-dihydroxypurine, and hypoxanthine with the time of hypoxia. The authors suggest that these compounds should be used as early markers for the diagnosis of hypoxia-ischemia in neonates [18].

An approach focusing on the analysis of metabolic changes in neonatal blood through the use of liquid chromatography coupled with mass spectrometry (HPLC-MS) represents a promising strategy to improve the neonatal diagnosis and prognosis of HIE. Given the complexity of this multifactorial and variable clinical condition, conventional biomarkers have limited efficacy [19]. Differential diagnosis of HIE requires identification of a specific pattern of biological molecules, which can be achieved by mass spectrometric methods. These analytical techniques facilitate the comprehensive analysis of several classes of molecules, including proteins, lipids, and metabolites. Taken together, these advances promise to optimize the diagnosis, treatment, and prognosis of neonates affected by HIE.

The aim of this study was to investigate and identify lipid markers in dried blood spots (DBSs) to accurately reflect the pathological changes in the brain caused by hypoxic-ischemic brain damage in 7-day-old rats.

## 2. Materials and Methods

### 2.1. Induction of Neonatal HIE Animal Model

The animal protocols used in this work were evaluated and approved by the institutional animal ethics committee according to FELASA guidelines. Experiments were performed on outbred white rats. Animals were obtained from the animal facility of the A.N. Belozersky Institute of Physico-Chemical Biology. The dams and their pups were kept in cages in a temperature-controlled environment (21 ± 2 °C), with lights on from 9:00 to 21:00. The dams had ad libitum access to food and water, and pups were examined daily for health status.

The modified Rice–Vannucci rat model for hypoxic-ischemic brain injury was used [20]. Neonatal hypoxic-ischemic injury was induced in postnatal seven-day-old pups of both sexes. Rats were first anesthetized with 1% isoflurane, then the left carotid artery was surgically isolated and electrocauterized. Hypoxia was then induced after 1.5 h with a gas mixture of 8% oxygen and 92% nitrogen for a period of 2 h in a multigas CO_2_ incubator (Binder, Tuttlingen, Germany) at a temperature of 37 °C. The mortality rate after hypoxia-ischemia induction was observed to be 3–5%. Pups from three litters were randomly divided into the following experimental groups: control (n = 16), intact rats; (2) HIE (n = 10), animals with HIE.

Four hours following the hypoxic exposure, magnetic resonance imaging (MRI) was performed to identify morphological changes in the brain regions affected by the damage in the rats. Six hours after the hypoxic exposure, dry blood spots were obtained for metabolomic analysis using high-performance liquid chromatography (Ultimate 3000 LC System) coupled with mass spectrometric detection (Maxis Impact qTOF) (HPLC-MS). A total of 26 samples were collected on FTA cards, and then three pellets with a diameter of 2 mm were extracted from each sample for analysis.

### 2.2. MRI Examinations of Brain Injury

Infarct volume was quantified by analyzing MR images of the brain obtained 4 h after hypoxia-ischemia as previously described [21] on a 7T magnet (Bruker BioSpec 70/30 USR, Bruker BioSpin, Ettlingen, Germany) with an 86 mm volume resonator for radiofrequency transmission and a phased-array mouse-head surface coil for reception. Before scanning, pups were anesthetized with isoflurane (2% induction, 1.5% maintenance) in a mixture of oxygen and air. Rats were placed in a prone position on a water-heated bed. The heads of the rats were fixed with a nasal mask and adhesive tape. The imaging protocol included a T2-weighted image sequence (time to repetition = 4500 ms; time to echo = 12 ms; slice thickness = 0.8 mm). For morphometric analysis of the MR images, we used ImageJ 1.53t software (National Institutes of Health, Bethesda, MD, USA). This allowed us to accurately calculate the infarct volume.

### 2.3. Dried Blood Spot Lipidomic Profiling by HPLC-MS

Metabolites were extracted from dry blood spots using the Folch method as described in the corresponding protocol [22]. To each sample, we added 480 µL of a chloroform–methanol mixture (i2:1 ratio) and 250 µL of water. The extraction mixture was then shaken vigorously for 10 min followed by centrifugation at 15,000× *g* for 10 min. We then collected 150 mL of the organic phase at the bottom in a separate tube. This extraction process was repeated with the addition of another mixture of chloroform–methanol and water. The supernatant from this second organic phase was combined with the previously collected one. The resulting mixture was dried with nitrogen at room temperature and then redissolved in 100 mL of an acetonitrile–isopropanol mixture (1:1 ratio).

High-performance liquid chromatography coupled with mass spectrometry (HPLC-MS) was conducted using an Atlantis T3 C18 column (3 µm, 15 cm in length, with an inner diameter of 1 mm, Waters, Milford, MA, USA) and the Ultimate 3000 Nano LC chromatographic system (Thermo Scientific, Waltham, MA, USA) [23,24]. The sample components were eluted using reverse-phase chromatography. Mobile phase “A” consisted of a mixture of ACN:H20 (60:40), while mobile phase “B” was composed of a mixture of IPA:ACN:H_2_O (90:8:2). Both phases contained modifiers (0.1% formic acid and 5 mm ammonium formate). Elution was performed with a gradient of mobile phase “B” at a flow rate of 40 µL/min: 0–0.5 min at 10%, followed by a 20 min gradient from 10% to 99%. Subsequently, a 10 min wash was performed with 99% of phase “B”, after which the phase was restored to its initial concentration (10% of phase “B”) within 1 min, and the column was balanced for 3 min. The total chromatography time for each sample was 34.5 min.

Metabolites were detected using a hybrid quadrupole time-of-flight mass spectrometer, the Bruker MaXis Impact (Bruker Daltoniks, Bremen, Germany), with two replicates per sample. Mass spectra were acquired at 50,000 resolution in the range of 50–1500 m/z in both polarities. Peak detection, grouping, and retention time correction were accomplished using the xcms software package. For peak detection, the Centwave algorithm was used with specific parameters: a maximum m/z deviation of 15 ppm and a minimum-to-maximum peak width of 10 or 45 s. Peak grouping across all samples was performed using the Peak Density method with default parameters [25,26]. For the identification of metabolites, we relied on the Human Metabolome Database (HMDB), which provided molecular weight matches (www.hmdb.ca, accessed on 5 June 2023).

### 2.4. Statistical Analysis

To identify and visually represent the differences between the groups, we applied the method of multivariate statistics, specifically the discriminant analysis of orthogonal partial least squares (OPLS-DA). In addition, we conducted univariate statistical analyses to assess the statistical significance of differences in relative concentrations (average integrated peak areas) of specific metabolites between groups using the *t* test. A *p* value less than 0.05 was considered statistically significant. Furthermore, we used a fold-change threshold of at least 1.5 as an additional criterion for identifying potential biomarkers.

To assess the statistical significance of the identified metabolic pathways, we applied Fisher’s exact test. All statistical and fold-change analyses were performed using the Metaboanalyst v5.0 platform.

## 3. Results

### 3.1. Evaluation of Brain Morphological Alterations

**Figure 1:**
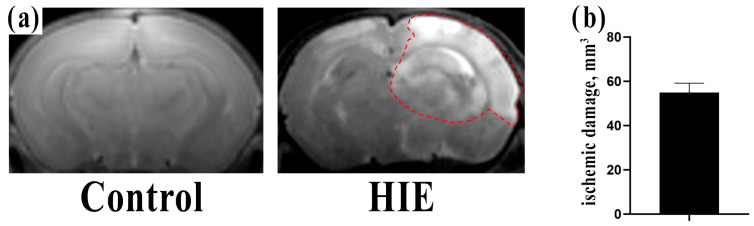
**Assessment of the extent of brain damage**. (**a**) Representative T2-weighted MR images of animal brain slices taken four hours after simulation of HIE. The damaged area is outlined with a dotted line. (**b**) Morphometric analysis of the volume of brain damage based on the MR images in the HIE group.

To identify lipidomic markers exclusively indicative of pathological brain changes following HIE, we used the Rice–Vannucci method to induce brain damage in vivo in 7-day-old rats of both sexes [20]. By analyzing T2-weighted magnetic resonance images, we found that simulated HIE resulted in impairments in the cortex, striatum, and hippocampus (as shown in Figure 1a). The damage volume in the ipsilateral hemisphere averaged 54.8 ± 4.3 mm^3^ (as shown in Figure 1b).

The time of blood sampling (6 h after hypoxic-ischemic injury) is the limit of the decision interval for assigning therapeutic hypothermia, the only approved treatment for HIE in neonatal practice. To identify potential markers of low-molecular-weight brain damage in blood, lipidomic HPLC-MS analysis was performed in two modes on 26 samples (10 from the HIE group and 16 from the control group) with subsequent identification of potential HIE markers.

### 3.2. DBS Metabolomic Profile in Positive Ion Mode

**Figure 2:**
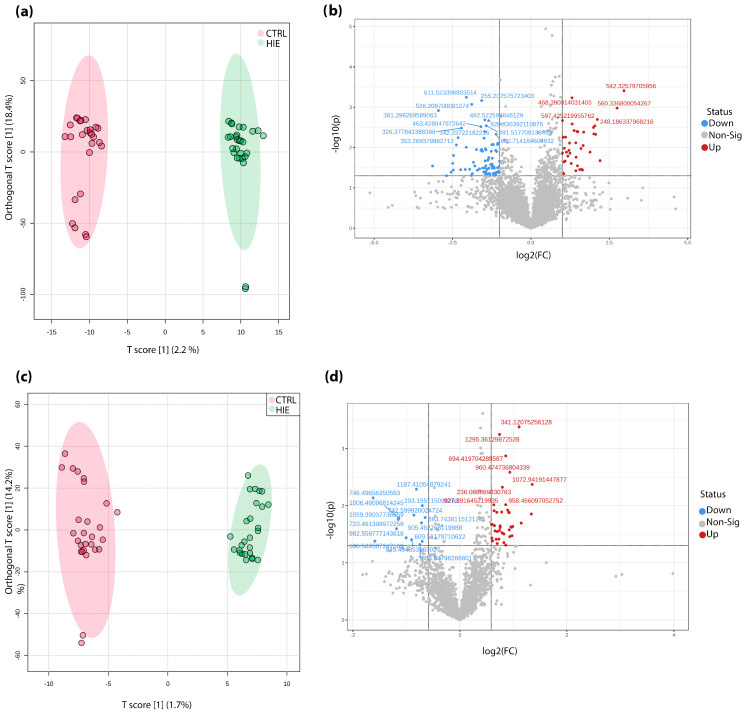
Comparative lipidomic analysis of DBS from HIE and control groups in both positive (**a**,**b**) and negative ion modes (**c**,**d**): OPLS analysis score plots and volcano plots.

In the first phase of the analysis, a large number of molecular ions, 9279 in total, were identified by a robust peak detection process. These molecular ions serve as indicators of the various compounds present in the samples. To conduct a systematic examination of the sample clustering and possible outliers, we used partial least squares discriminant Analysis (OPLS-DA), a statistical method for strong predictive modeling and variable selection capability. The analysis revealed a distinct separation along the first predictive component, effectively distinguishing the ischemia-hypoxia samples from the control group (Figure 2a).

**Figure 3:**
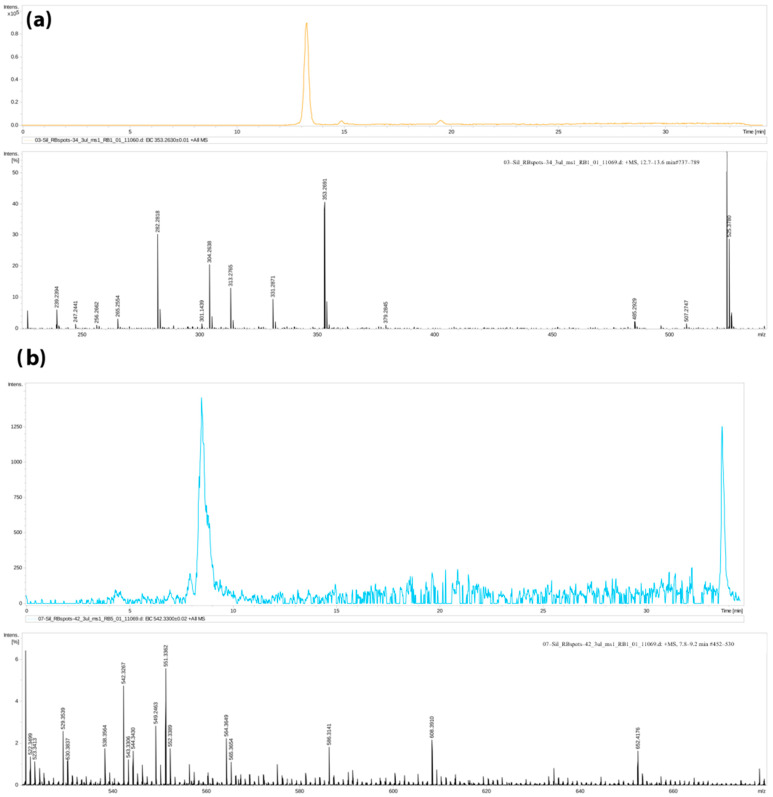
Examples of chromatograms and MS spectra for potential markers of hypoxic-ischemic damage: (**a**) lysophosphatidylcholine, lysoPC(20:5), and (**b**) monoacylglyceride, MG(16:0).

Molecular ions that showed statistically significant differences (*p* < 0.05) with a fold change of ≤1.5 between the HIE and control groups were considered as potential biomarkers. A total of 120 prospective candidates were identified (see Figure 2b and Supplementary Table S1). The HMDB metabolite database was employed for identification purposes. Table 1 lists some of the most distinctive compounds. Within the hypoxia-ischemia group, we observed a notable decrease in blood phenylalanine (by a factor of 6.5), N-a-Acetyl-L-arginine, and steroid hormones such as 18-oxocortisol and pregnanediol. At the same time, there was a decrease in saturated mono- and triacylglycerides (MGs, TGs) and phosphatidylcholines (PCs), whereas unsaturated PCs increased in the HIE group. Figure 3 shows examples of chromatograms and mass spectra for these compounds for illustration.

### 3.3. DBS Metabolomic Profile in Negative Ion Mode

In negative ion mode, our HPLC-MS detection identified a total of 3545 molecular ions. The results of the OPLS-DA analysis are shown in Figure 2b. It is worth noting that the differences between the groups on the primary component are less pronounced compared to the positive ion mode, but the group clustering remains evident. Performing univariate statistical analysis, we identified 54 molecular ions that exhibited statistically significant changes (*p* < 0.05), as shown in Figure 2d and Supplementary Material Table S1. The results of the identification of these potential markers are given in Table 1. In particular, lipids associated with TG, phosphatidylinositols (PIs), and PCs were among the most significantly altered ions in the HIE group.

### 3.4. Metabolic Pathways Involved in the Development of Hypoxia-Ischemia

Metabolic pathway enrichment analysis was conducted using data from HMDB and the Metaboanalyst v.5.0 platform. For this analysis, we utilized the molecular ions that made the most substantial contributions to the differences between the groups, as indicated by the OPLS analysis. The results of the metabolic pathway enrichment analysis, revealing the pathways with significant changes, are shown in Table 2. Of note, we observed striking changes in the steroid biosynthesis pathway in both positive and negative ion modes. This finding highlights the potential importance of steroids in the context of ischemic-hypoxic brain injury. In addition, the identified metabolites play a significant role in five specific signaling pathways, namely retinol metabolism, arachidonic acid metabolism, linoleic acid metabolism, unsaturated fatty acid biosynthesis, and phospholipid metabolism.

## 4. Discussion

Timely recognition of neonates at high risk for developing moderate or severe HIE in the critical first hours after birth is critical for optimal management and treatment [27]. However, conventional clinical and laboratory tests often quickly revert to normal values, making it difficult to accurately assess the duration of asphyxia-ischemia and the extent of brain damage caused by HIE [28,29,30]. Although advanced imaging modalities such as MRI, amplitude-integrated electroencephalography, and multichannel electroencephalography have high prognostic potential and are routinely used in leading perinatal centers, their use is usually limited to the first six hours of life, when the decision to initiate therapeutic hypothermia must be made [31,32,33,34]. This presents a significant clinical hurdle because therapeutic hypothermia, a recognized intervention for HIE, is most effective when initiated immediately within the critical time window. Given the limitations of currently available diagnostic tools in the first hours of life, it is becoming increasingly urgent to explore new HIE markers using advanced “omics” approaches. These advanced methods, such as metabolomics, genomics, proteomics, and transcriptomics, offer the potential to identify specific biomarkers associated with the early stages of HIE [35].

The metabolic profile of blood serves as a real-time reflection of cellular and physiological changes in metabolism [36]. Model experiments offer the advantage of establishing precisely controlled conditions to identify potential markers associated with specific molecular processes in response to various stimuli, particularly in the context of hypoxia-ischemia [37,38,39]. HIE is characterized by compromised integrity of the blood–brain barrier, which may result in the appearance of brain injury markers in the peripheral bloodstream [40]. Experimental in vivo models of hypoxic-ischemic brain injury in rats, mice, and primates are actively used to identify potential metabolomic biomarkers, but the effects of hypoxia-ischemia on blood lipid profiles have not been investigated so far [41,42]. Using UPLC-MS analysis, the lipidomic profile of DBS obtained from neonatal rats can provide valuable insights into novel metabolic pathways that reflect an adaptive response to acute hypoxia. Furthermore, this analysis may reveal early biomarkers that facilitate the diagnosis of birth asphyxia and allow the prediction of its neurological consequences. We chose the widely used and well-characterized Rice–Vannucci model of neonatal hypoxia-ischemia. It accurately depicts the pathogenesis of perinatal asphyxia and establishes a clear cause-and-effect relationship between hypoxia-ischemia and brain injury and allows for the identification of biomarkers specifically associated with this condition [43,44].

DBSs have been actively used in newborn metabolic screening programs for over 50 years [45,46]. The attractiveness of the secondary use of DBSs in the search for neonatal biomarkers is the small amount of blood required, simplicity, high reproducibility, and inexpensive procedure of the blood collection process, as well as the ease of transportation and storage [47]. These samples are particularly attractive when serial sample collection is required. The use of modern technologies such as HPLC-MS and nuclear magnetic resonance allows the simultaneous analysis of hundreds to thousands of compounds in a minimal sample volume [48].

In this study, for the first time, an in-depth analysis of the primary metabolic changes of whole blood (dry spots) in a model experiment of hypoxic-ischemic injury, focusing on the lipid spectrum, was performed using HPLC-MS. The data obtained were analyzed to identify potential low-molecular-weight markers of brain damage. Multidimensional PLS-DA analysis of chromatography–mass spectrometry data of the organic extract of DBS from 7-day-old rats 6 h after induced hypoxia-ischemia using the Rice–Vannucci method showed clear separation of experimental groups. The data obtained indicate a clear change in the composition of whole blood metabolites under the influence of ischemia/hypoxia in rat pups. One-dimensional statistical analysis was used to select and identify molecular ions whose concentration changed statistically significantly between the groups and to analyze the metabolic pathways in which these metabolites are involved. Thus, the analysis of dry spots identified a decrease in the level of monoacylglycerides; an increase in diacylglycerides and lysophosphatidylcholines; and multidirectional changes in triacylglycerides, phosphatidylcholines, phosphatidylethanolamines, and phosphatidylinositols.

The metabolism of glycerophospholipids was found to be one of the most involved processes in the development of a systemic response to hypoxia-ischemia. This metabolic pathway includes a wide range of major classes, including monoacylglycerides, diacylglycerides, triacylglycerides, phosphatidylcholines, phosphatidylethanolamines, and phosphatidylinositols, as well as their derivatives such as oxidized lipids, plasmalogens, and lysophosphatidylcholines. Essentially, these pathological changes affect the entire lipid spectrum of the blood, which is consistent with previous research findings [49]. The disturbances in the lipid composition of the blood are closely related to alterations in the biosynthesis of unsaturated fatty acids (*p* < 0.0001) and in the metabolism of arachidonic acid (*p* < 10^−6^) and linoleic acid (*p* < 10^−5^). Arachidonic acid, an omega-6 polyunsaturated fatty acid (AA, C20:4), is synthesized in the body from essential linoleic acid (C18:2). It plays a crucial role as a major component of phosphatidylethanolamines, phosphatidylcholines, and phosphatidylinositols in the cell membranes of the brain, muscles, and liver. In addition, AA is one of the most abundant fatty acids in the brain. Under conditions of hypoxia-ischemia, where cerebral blood flow is interrupted, oxygen-deprived brain tissue begins to rapidly cleave AA from the phospholipid bilayer of the membrane [50].

Under adverse conditions (such as cerebral hypoxia/ischemia, hemorrhagic stroke, trauma), extracellular glutamate accumulates, leading to a toxic increase in nitric oxide and reactive oxygen species (ROS). This, in turn, leads to a substantial influx of extracellular calcium ions into brain cells and the release of Ca^2+^ from intracellular stores [51]. Increased intracellular Ca^2+^ levels activate membrane phospholipases (PLA2s), proteases, and nucleases [52]. Hydrolysis of membrane phospholipids by PLA2s leads to the release of free fatty acids (especially AA), lysophosphatidylcholines, diacylglycerides, eicosanoids, lipid peroxides, and free radicals [53]. In this experimental study, a significant increase in lysophosphatidylcholines, lysoPC(18:3), and lysoPC(20:5) (by more than 3.9-fold) was observed in DBS, which is consistent with the results of other authors [54]. Lysophosphatidylcholines are potent mediators of inflammation in the brain due to the stimulated release of interleukin-1β and subsequent activation of microglia [55,56]. In addition, the rapid accumulation of free AA during ischemic or hemorrhagic brain injury also increases the level of oxidative stress [57]. The uncontrolled oxidation of AA, known as the “arachidonic acid cascade”, results in high levels of prostaglandins, leukotrienes, thromboxanes, isoprostanoids, and nonenzymatic lipid peroxidation products [52,58]. Similarly, the negative effect of linoleic acid C18:2 and its oxidized metabolites on the development of ischemic brain damage has been demonstrated. Linoleic acid serves as a direct precursor of bioactive oxidized linoleic acid metabolites and also as a precursor of arachidonic acid [59,60].

Brain tissue is particularly susceptible to oxidative stress because it consumes large amounts of oxygen to meet high metabolic demands. Neuronal membrane lipids are rich in polyunsaturated fatty acids, which make them susceptible to peroxidative damage. In addition, the brain has lower levels of antioxidants compared to other organs [52]. The release of AA by PLA2s and its subsequent metabolism play an important role in the oxidative damage of nervous tissue after hypoxia, including subsequent reoxygenation [61,62,63]. In addition, ω-hydroxylation of arachidonic acid by cytochrome P450 4A and others produces a potent vasoconstrictor, 20-hydroxyeicosatetraenoic acid (20-HETE) [64,65]. Inhibition of 20-HETE in vivo results in less damage to cerebral vessels and improves outcomes after brain ischemia [66,67].

However, there is emerging evidence that prolonged intraperitoneal administration of AA protects the brain in an ischemia/reperfusion model induced by middle cerebral artery occlusion in rats by inhibiting inflammation, oxidative stress, and lipid peroxidation [68]. Moreover, the neuroprotective effect of lipoxin A4 (LXA4), a metabolite of AA, was described in a rat model of global cerebral ischemia. Intracerebroventricular injection of LXA4 suppressed acute inflammation and oxidative stress and prevented blood–brain barrier disruption by regulating the IκB/NF-κB pathway [69].

This study demonstrates a close relationship between hypoxic-ischemic brain damage and alterations in antioxidant metabolism, particularly in relation to retinol levels in peripheral blood. In addition, a significant decrease in plasma N-acetylarginine levels was observed in the HIE group. The neurotoxic effects of arginine (Arg) and its metabolites, including guanidine compounds such as N-acetylarginine, homoarginine, and argic acid, are well known. In oxidative stress, an increase in Arg and its metabolites can lead to increased production of nitric oxide (NO), increased free radical levels, impaired antioxidant protection (due to decreased activity of superoxide dismutase and glutathione peroxidase), oxidation of proteins, and induction of lipid peroxidation in cell membranes [70,71]. Elevated levels of ROS prove to be one of the most critical factors contributing to neuronal cell death [72]. Oxidative stress results from an imbalance between the production of ROS and the detoxification capacity of the biological system for reactive intermediates or efficient damage repair. Endogenous and exogenous antioxidants, together with antioxidant defense enzymes, play an essential role in protecting against oxidative stress and have the potential to act as neuroprotective agents [73,74]. An in vivo study conducted in mice suggested a protective mechanism of antioxidants against the deleterious effects induced by N-acetylarginine [75]. Significant changes in antioxidant metabolic pathways (with a *p*-value < 10 × 10^−11^) appear to be a protective response of the organism aimed at minimizing hypoxic-ischemic damage, particularly the reduction in ROS.

Moreover, the steroid hormone biosynthetic pathway was significantly altered during induced hypoxic-ischemic injury by both modes of analysis (with a *p*-value < 0.001). Our results are consistent with previous findings indicating a significant increase in steroid hormone levels in blood samples from neonates who had suffered asphyxia and neonatal convulsions at birth [76]. In the study by Piñeiro-Ramos JD et al., it was observed that the alteration in the steroid hormone biosynthetic pathway in neonates diagnosed with moderate/severe hypoxic-ischemic encephalopathy persisted throughout the observation period, which included the time from onset to completion of therapeutic hypothermia [77]. This suggests that steroid hormone dysregulation is persistent and not just a transient response to HIE. This may be related to the response to stress and pain, which inevitably trigger a hormonal response. In addition, hormones have been proposed as therapeutic agents in HIE. In experimental studies, administration of glucocorticoids and progesterone improved outcomes after hypoxic-ischemic brain injury in neonates [78,79].

In line with our results, human studies propose glycerophospholipids, lipid peroxidation (e.g., unsaturated fatty acids) products, as well as lactate, tricarboxylic acid cycle intermediates (like succinate and hypoxanthine), amino acids, and acylcarnitines, as potential markers of perinatal asphyxia [15,77,80,81,82]. However, the use of a single marker for the diagnosis of infants with significant perinatal asphyxia appears to have limitations [83,84]. Therefore, a metabolomic approach may provide a more reliable means of stratifying the risk of developing HIE. Thus, Reinke S.N.’s group developed and validated a metabolomics index based on levels of succinate, glycerol, β-hydroxybutyrate, and O-phosphocholine in umbilical cord blood [85,86]. A calculated value greater than 2.4 predicted severe neurodevelopmental abnormalities at 3 years with a sensitivity of 80% and a specificity of 100%. However, further studies with serial sampling are needed to understand the complexity and variability of HIE. In this context, DBS can be proposed as the most promising and convenient samples because they minimize the preanalytical steps and have been shown to closely resemble protein-precipitated plasma [87,88].

As a limitation of our study, it is important to mention that the control rats were not treated with isoflurane, unlike the group subjected to HIE. Isoflurane is known to have both neuroprotective and neurotoxic properties, and the effects depend on the duration of inhalation and the concentration of the anesthetic [89,90,91]. In our study, pups were anesthetized for a relatively short time during common carotid artery ligation, 5–7 min on average. We believe that the use of isoflurane in this manner resulted in minimal side effects, including possible neuroprotection. It should be noted, however, that despite the potential neuroprotective effect of isoflurane, our analysis of T2-weighted images 4 h after HIE revealed a significant area of damage with an average volume of 54.8 ± 4.3 mm^3^, indicating substantial brain injury. It can be hypothesized that similar markers might have higher concentrations in children without the influence of isoflurane. In addition, the toxic effect of isoflurane can be excluded in our study because usually it is observed only with very long inhalation (more than 4 h) [89].

Another limitation of our study is the potential systemic effects of hypoxia on other organs and tissues, which may also alter the metabolic profile studied. It is important to emphasize that our experimental design aimed to mimic brain injury by inducing both systemic hypoxia and cerebral ischemia by unilateral carotid artery transection. The combination of these two factors was necessary to observe significant brain injury visible on T2-weighted magnetic resonance images. Previous studies by our group have shown that hypoxia alone does not lead to the formation of focal brain lesions [92]. Although we cannot rule out a systemic effect of hypoxia on other organs and tissues, it is important to remember that the brain is the organ most sensitive to ischemic impact. Therefore, we believe that mainly hypoxic-ischemic brain damage is responsible for the identification of metabolites in our study.

## 5. Conclusions

Metabolic profiles in blood reflect changes in cellular metabolism in real time and provide insights into adaptive responses to acute hypoxia. DBSs are a convenient and cost-effective method for biomarker analysis because they require small volumes, are easy to transport and store, and allow for serial sampling in neonatology. In-depth HPLC-MS analysis of the lipid spectrum in whole blood samples derived from a model experiment with hypoxic-ischemic injury provides the opportunity to identify specific low-molecular-weight markers associated with brain injury. Further research in this area could lead to improved diagnostic tools and prediction of the neurological consequences of birth asphyxia.

## Figures and Tables

**Table 1:** Potential DBS markers of hypoxic-ischemic damage with a change ≤ 1.5 in the HIE group compared with the control (*p* < 0.05). MG—monoacylglyceride, TG—triacylglyceride, PC—phosphatidylcholine, PE—phosphatidylethanolamine, PA—phosphatidic acid, Ox—oxidized lipids, lysoPC—lysophosphatidylcholine, plasmanyl-PC—plasmanylphosphatidylcholine, plasmenyl-PC—plasmenylphosphatidylcholine, PE-Nme—monomethylphosphatidylethanolamine, PI—phosphatidylinositol, PGP—phosphatidylglycerophosphate, PIP—phosphoinositide.

m/z	Metabolite	Adduct	Fold Change	*p*-Value
166.084	Phenylalanine	M + H	−6.46	0.5
377.1911	18-Oxocortisol	M + H	−5.53	0.02
433.3341	MG(22:2)	M + Na	−5.19	0.01
353.2694	MG(16:0)	M + Na	−5	0.01
611.5234	TG(34:0)	M + H	−4.17	0
217.1292	N-a-Acetyl-L-arginine	M + H	−3.56	0.02
578.4194	LysoPC(22:1)	M + H	−2.84	0.04
381.2952	MG(18:0)	M + Na	−2.71	0.04
744.555	PE(36:2)	M + H	−2.36	0.03
913.7763	OxTG(58:10)	M + H	−2.35	0.02
880.7142	Plasmenyl-PC(42:0)	M + Na	−2.16	0.01
343.2658	Pregnanediol	M + Na	−2.14	0.04
700.4877	PE-Nme(30:0)	M + Na	2.01	0.01
792.5673	OxPC(34:1)	M + H	2.04	0.01
506.3589	Lyso-Plasmenyl-PC(18:1)	M + H	2.15	0.01
667.5317	DG(38:4)	M + Na	2.27	0.02
898.5567	OxPE(44:8)	M + Na	2.31	0.01
823.5297	PA(44:8)	M + Na	2.72	0.02
865.7681	OxTG(54:6)	M + H	2.77	0
518.3246	LysoPC(18:3)	M + H	3.99	0
542.3258	LysoPC(20:5)	M + H	7.82	0
293.16	Tetradecanedioic acid	M + Cl	−1.63	0.01
609.51	TG(34:0)	M − H	−1.63	0.04
694.42	PE(30:2)	M + Cl	1.81	0
746.50	OxPE(34:2)	M − H	−3.08	0.01
834.49	PC(38:9)	M + Cl	1.68	0.02
845.49	PI(32:0)	M + Cl	−1.87	0.04
852.50	OxPC(38:8)	M + Cl	1.86	0.01
853.50	PGP(36:2)	M − H	−1.87	0.03
879.74	TG(54:5)	M − H	1.98	0.02
893.74	TG(52:2)	M + Cl	−1.57	0.02
905.46	OxPI(36:6)	M + Cl	1.64	0.02
913.48	PIP(34:2)	M − H	1.60	0.04
914.68	Plasmalyl-PC(44:4)	M + Cl	1.61	0.01

**Table 2:** Metabolic pathways most likely affected by neonatal ischemia-hypoxia (*p* < 0.05).

Pathway Name	*p*-Value
Positive ion mode	
Retinol metabolism	7.9 × 10^−12^
Arachidonic acid metabolism	8.8 × 10^−7^
Linoleic acid metabolism	4.7 × 10^−6^
Unsaturated fatty acid biosynthesis	9.7 × 10^−5^
Phospholipids metabolism	0.005
Steroid biosynthesis	0.04
Negative ion mode	
Steroid biosynthesis	6.5 × 10^−4^
Bile acid biosynthesis	0.035

## Data Availability

Data are contained within the Supplementary Material.

## Supplementary Materials

The following supporting information can be downloaded at https://www.mdpi.com/article/10.3390/children10101693/s1.
